# Supplementary material for: Modification of Poly(4-methyl-2-pentyne) in the Supercritical Fluid Medium for Selective Membrane Separation of CO2 from Various Gas Mixtures
Source: Polymers (Basel). 2020 Oct 24;12(11):2468. doi: 10.3390/polym12112468 (PMC7693599; doi:10.3390/polym12112468)
Supplement: Supplementary file 1 [file polymers-12-02468-s001.pdf]

Supporting Information for the manuscript:

Modification of poly(4-methyl-2-pentine) in the supercritical fluid medium for selective membrane separation of CO_2 from various gas mixtures

Viktoriya Polevaya[1],* Anton Vorobei[2], Andrey Gavrikov[2], Samira Matson[1], Olga Parenago[2], Sergey Shishatskiy[3,*] and Valeriy Khotimskiy[1]

1 A.V. Topchiev Institute of Petrochemical Synthesis RAS, 119991, Leninsky pr., 29, Moscow, Russian Federation; tips@ips.ac.ru

2 Kurnakov Institute of General and Inorganic Chemistry RAS, 119991, Leninsky pr., 31, Moscow, Russian Federation; info@igic.ras.ru

3 Institute of Polymer Research, Helmholtz-Zentrum Geesthacht, Max-Planck-Strasse 1, 21502 Geesthacht, Germany; contact@hzg.de

* Correspondence: polevaya@ips.ac.ru, Tel +7 495 647 5927 ext.205; sergey.shishatskiy@hzg.de, Tel.: +49 4152 87 2467

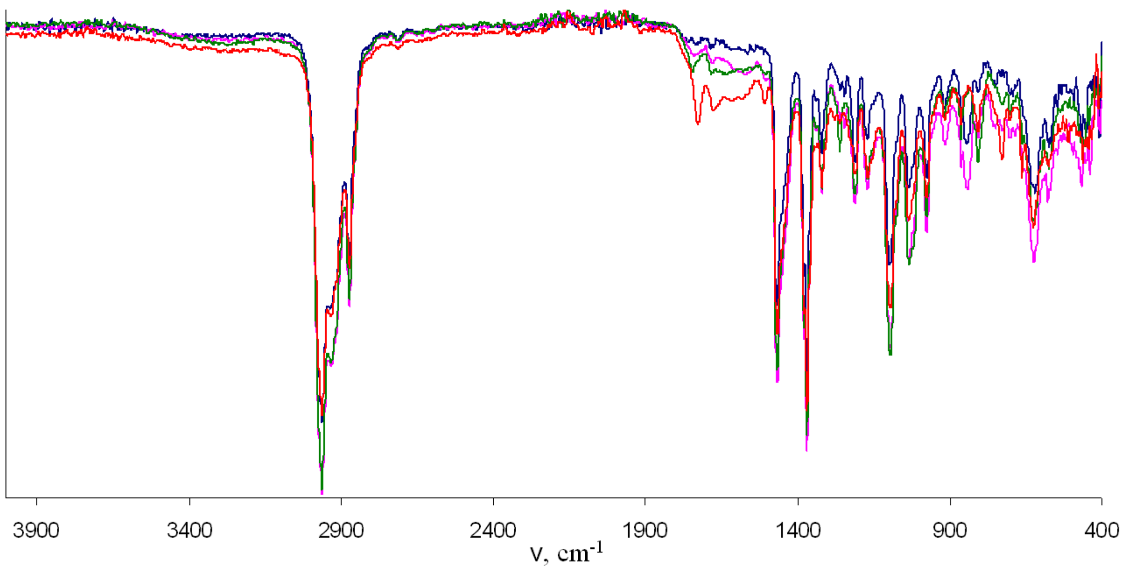

Figure S1. IR spectra of PMP-Br (blue line), Samples: 1 (pink line), 2 (green line) and 3 (red line).

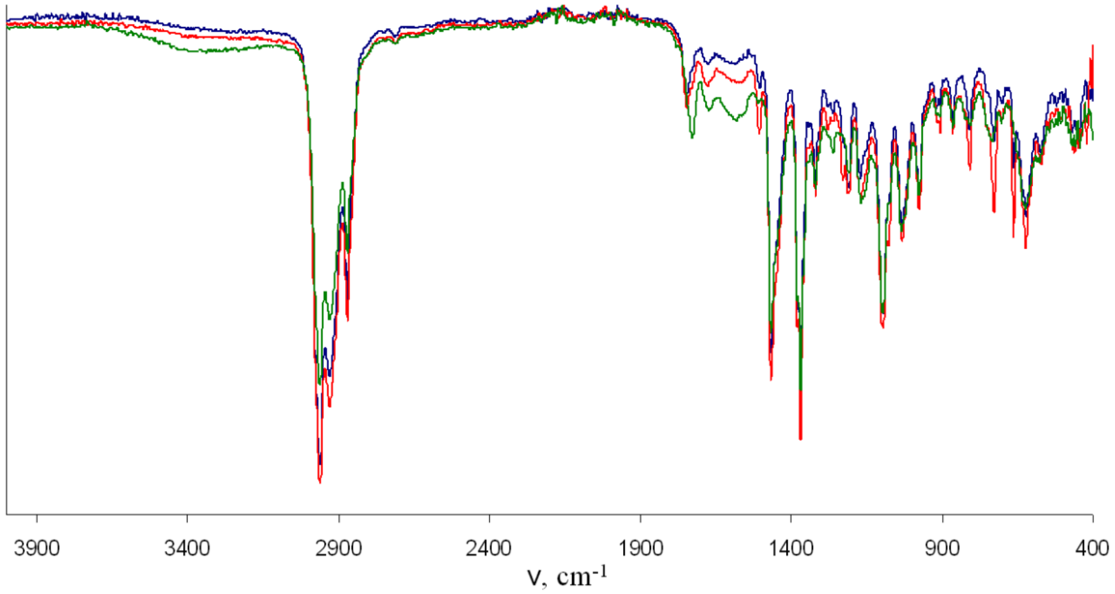

Figure S2. IR spectra of the Samples: 4 (blue line), 5 (red line) and 6 (green line).

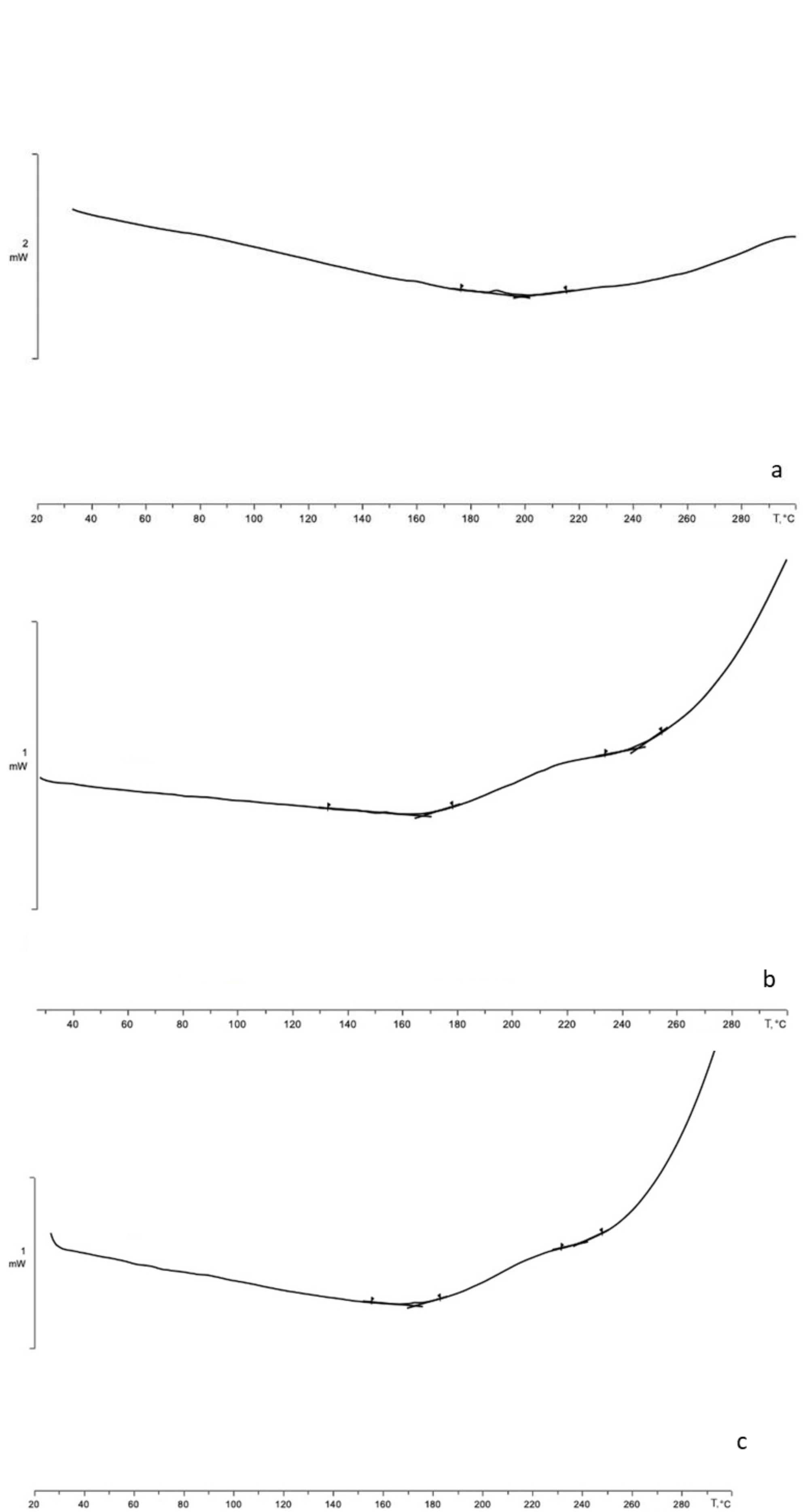

Figure S3. Examples of DSC measurements for samples: PMP-Br (a), samples 3 (b) and 6 (c).

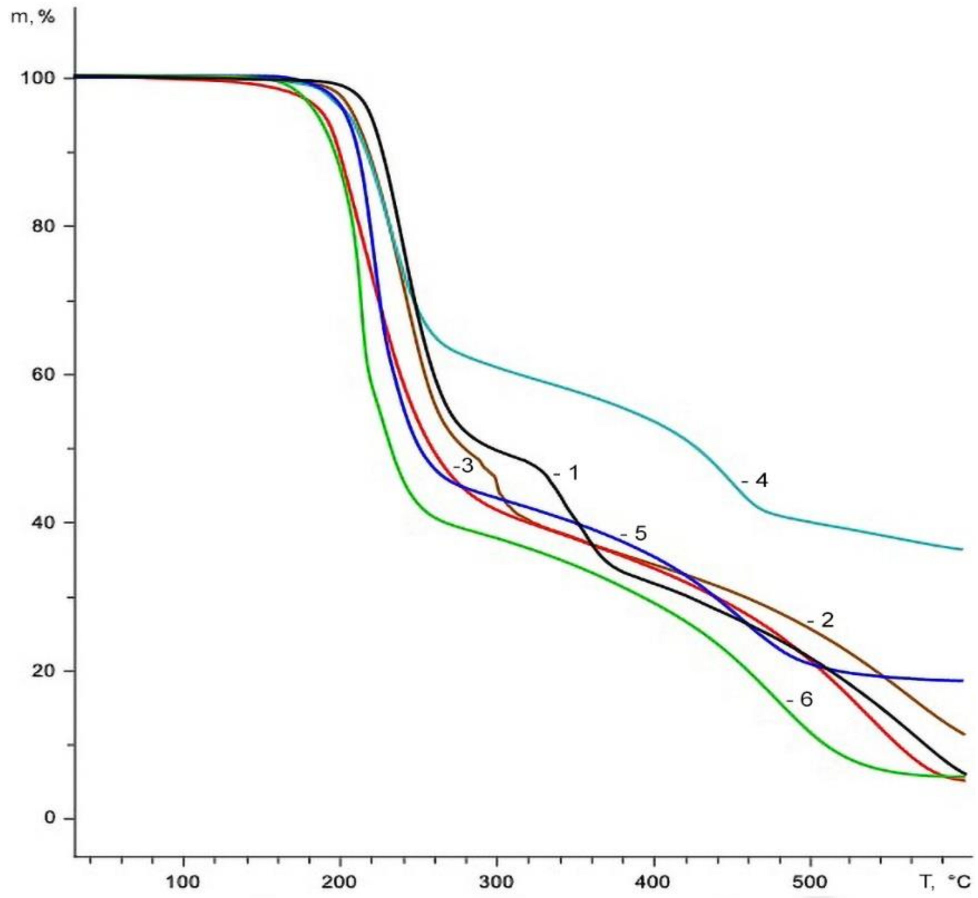

Figure S4. Results of TGA investigation of quaternized samples 1-6. The curve for the initial PMP can be found in the main text, Figure 8.
